# 

**Published:** 1905-09-15

**Authors:** 


### CONTENTS

Event and Comment - - - - - - - - - - 433

The Importance of Systematic Training in Operative Technics

By Fred. W. Gethro, D.D.S. 439

The Advantages Of Nitrous Oxid Gas and the Proper Manner of Administering It, - - - - By S. Strauch, D.D.S. 448

Reasons for Preferring Somnoform to Nitrous Oxid as a General Anesthetic, - - - - - - - By [illegible] Bowles 460

The Cement Problem, - - - By G. C. Poundstone, D.D.S. [illegible]

Obituary - - - - - - - - - - - 479

Indents - - - - - - - - - - - - - 481

Announcements - - - - - - - - - - 484

